# School Staff Perspectives on Tobacco Prevention: Findings From a Statewide Educator Survey

**DOI:** 10.1177/10901981251391146

**Published:** 2025-11-15

**Authors:** Joanna Sun, Gino Acevedo, Ting Luo, Ding Ding Wang, Christopher M. Anderson, Shu-Hong Zhu

**Affiliations:** 1 University of California San Diego, La Jolla, CA, USA

**Keywords:** tobacco, prevention, education, school health, school staff training, school-based interventions

## Abstract

The California Department of Education (CDE) has operated the statewide, school-based, Tobacco-Use Prevention Education (TUPE) Program since 1989. Evaluating the impact of such a program is challenging in a real-world context, where multiple tobacco prevention activities have been ongoing for years and occur simultaneously. This study aimed to determine how TUPE funding was distributed geographically and whether it was associated with increased tobacco training and prevention activity in California public schools. TUPE funding levels were compared by region, and staff of randomly selected middle and high schools (*n* = 6,115 staff from 1,383 schools) were recruited to participate in the California Educator Tobacco Survey, administered March–June 2022. TUPE-funded and non-TUPE-funded schools were compared on respondent characteristics, health priorities, substance use prevention training, and tobacco prevention activities. Regional distribution of funding followed the same rank order as youth tobacco use rates. TUPE-funded staff were significantly more likely than non-TUPE-funded staff to perceive tobacco as a school health priority (28.0% vs. 22.6%), receive training on tobacco prevention (odds ratio [OR] = 1.4), and report that their school offered >3 tobacco prevention activities (OR = 2.1). The main conclusion was that TUPE funding was associated with increased tobacco training and prevention activity. The study also demonstrated the value of school staff surveys in assessing the implementation of tobacco prevention programming. Encouraging applications from rural, high-prevalence areas may be important in countering geographic disparities. To address the low prioritization of tobacco, future tobacco prevention efforts may aim for closer integration with other student health programs (e.g., mental health, marijuana use prevention).

California has for many years provided school-based tobacco prevention programming. In 1988, the state’s voters passed Proposition 99, a tobacco tax initiative establishing the first comprehensive tobacco control program in the U.S. (Roeseler & Burns, 2010). As part of this effort, the California Department of Education (CDE) established the statewide, school-based, Tobacco-Use Prevention Education (TUPE) program (California Department of Education, Whole Child Division, Tobacco-Use Prevention Education Office 2025). Due in part to the success of tobacco programming in California, tax revenues from Proposition 99 dwindled significantly over time, making it more difficult to support TUPE programming across the state, especially in rural areas with smaller student populations. However, in 2016 California voters passed Proposition 56, which increased the cigarette tax by $2 per pack and added equivalent taxes on vapes and other tobacco products (Ballotpedia, 2016), leading to an infusion of new funding for tobacco prevention.

TUPE supports local tobacco prevention programming via four main types of grants: (1) American Indian Education Center grants to help reduce commercial tobacco use among American Indian youth; (2) County Technical Assistance grants to fund county-level TUPE programming; (3) Tier 1 grants to fund basic district-level TUPE programming, including the implementation of tobacco-free school policies and youth tobacco surveillance; and (4) Tier 2 grants to fund more comprehensive TUPE programming (California Department of Education, Whole Child Division, Tobacco-Use Prevention Education Office, 2025). Tier 2 grants are competitively awarded and account for the bulk of TUPE spending. Tier 2 grantees perform the same policy and surveillance work as Tier 1 grantees, but they also implement evidence-informed tobacco prevention curricula for students in grades 6–12, provide intervention and cessation services for students who use tobacco, and provide tobacco-related youth development opportunities (California Department of Education, Tobacco-Use Prevention Education Office, 2020). For the 2020–2021 school year, the first in a three-year grant cycle, Tier 2 grantees were awarded nearly $24 million (California Department of Education, 2021).

Guidelines, reviews, and other works have long been available to help guide the development and implementation of school-based tobacco prevention programs. In 1989, an expert advisory panel convened by the National Cancer Institute described the essential elements of such programs (Glynn, 1989). In 1994, the Centers for Disease Control and Prevention published guidelines on preventing tobacco use and addiction through school health programs (Centers for Disease Control and Prevention, 1994; McCormick & Tompkins, 1998). More recently, the Substance Abuse and Mental Health Services Administration (SAMHSA) released a resource guide on evidence-based practices for reducing vaping among youth and young adults (SAMHSA, 2020). Multiple systematic reviews have been published, generally finding these programs to be modestly effective (Flay, 2009; La Torre et al., 2005; Sherman & Primack, 2009; Thomas et al., 2013, 2015). The U.S. Surgeon General concluded that they can have at least short-term effects but are more likely to have sustained effects when implemented in the context of comprehensive tobacco control programs (U.S. Department of Health and Human Services, 2012). Various curricula have been disseminated to help schools address adolescent smoking and, more recently, vaping (Baker et al., 2022; Gaiha et al., 2021; Liu et al., 2020, 2022; McCauley et al., 2023).

Despite the available evidence and resources, evaluating the impact of a particular school-based program such as TUPE is challenging. In the real-world context, where multiple tobacco prevention and control activities have been ongoing for years and still occur simultaneously, it is difficult to detect additional effects from a school-based program. Moreover, such programs tend to be implemented over large populations in multiple settings, with varying degrees of fidelity to curriculum. Regardless, in a well-funded program such as TUPE, it should be possible to detect at least some effects. Evaluation of TUPE relies primarily on two biennial surveys. The first, conducted with school staff, assesses staff perspectives on tobacco prevention programming and intermediate outcomes indicating degree of program implementation. The second, conducted with students, assesses student attitudes and behavior relating to tobacco use. This study focuses on the former.

The study aimed to answer three questions: (1) How well was TUPE funding distributed across California, including in regions with higher youth tobacco use rates? In other words, was funding allocated where tobacco use prevalence was greatest? The TUPE Office actively encouraged grant applications from rural and other high-prevalence areas of the state, and it was expected that this would be reflected in the final distribution of funding. (2) Were staff from TUPE-funded schools more likely to receive tobacco training than staff from non-TUPE schools? (3) Did TUPE-funded schools deliver more tobacco prevention activities than non-TUPE schools? If funding was used as intended, there should be differences in the likelihood of staff receiving training and in the number of tobacco prevention activities offered. To answer the first question, we examined records of TUPE Tier 2 awards and compared funding levels by region in reference to youth tobacco use rates by region. We focused on Tier 2 TUPE because only these schools received funding to conduct tobacco education activities. For the other two questions, we surveyed more than 6,000 staff from 1,349 middle and high schools across California to determine whether TUPE funding was associated with an increased likelihood of receiving training and a higher number of tobacco prevention activities.

## Method

### Participants and Procedures

Participants were school staff of randomly selected public middle and high schools across California who took the California Educator Tobacco Survey (CETS), administered March–June 2022. CETS was developed through an iterative process involving feedback from community and scientific advisory boards and a thematic analysis of prior qualitative interviews which were used to inform survey items and response options. Survey topics included staff roles, school health priorities, training, anti-tobacco activities, and perceived impacts of school-based tobacco prevention efforts. The survey was administered online via the Qualtrics XM Platform and took approximately 15 minutes to complete.

Only traditional-format schools with at least 30 students in a middle or high school grade were sampled (California Department of Education, 2024). All high schools meeting this threshold were included. Middle schools were sampled in a 1-to-3 ratio (i.e., 1 middle school for every 3 high schools).

The initial sampling pool for CETS consisted of 2,008 schools, including 500 middle and 1,508 high schools. Online directories for these schools were scraped for the names, roles, and email addresses of staff. Prospective participants were those with roles likely to bring them into direct contact with students, including teachers, administrators, counselors, and school health professionals. Missing email addresses were formulated from staff names and standard email address patterns (e.g., jdoe@k12.org), where possible. During this process 307 schools were removed, either because it was impossible to find or generate emails for their staff or because the schools themselves turned out to be ineligible (e.g., virtual instruction only, home-based school). After adjusting for these anomalies, 97,833 invitations were sent to staff of 1,701 schools, including 438 middle schools and 1,263 high schools. Participation in the survey was incentivized with the chance to win one of multiple $100 gift cards, with 1-in-120 odds of winning.

Responses were received from 7,278 individuals from 1,383 schools, including 112 who declined to participate, 384 who were no longer affiliated with the invited schools, and 667 who completed less than half of the survey. These were excluded, leaving a final sample of 6,115 individuals from 1,349 schools. The individual-level response rate was 6.3%, with an average of 4.5 respondents per school. The school-level response rate was 79.3%. Many of the schools with no responses utilized spam filters or district blocks that prevented incoming email invitations from reaching their staff.

The study was approved by the University of California, San Diego, Human Research Protection Program (#803637).

### Measures

#### Tobacco Use Prevalence and Funding Information

The California Student Tobacco Survey (CSTS), administered during the 2019–2020 school year, was used to determine the prevalence of current tobacco use among public school students, defined as the percentage of students reporting use in the past 30 days. CSTS was a biannual survey of middle and high school students conducted by the California Department of Public Health as part of ongoing efforts to track statewide tobacco use trends (Zhu et al., 2020, 2021). The 2019–2020 survey was used because it had a much larger sample than the 2021–2022 survey (Dutra et al., 2023; Zhu et al., 2021). Publicly available enrollment data for the 2020–2021 school year (California Department of Education, 2024) and funding records for TUPE Tier 2 grants in the 2020–2023 funding cycle (California Department of Education, 2021) were used to calculate the proportion of students in TUPE-funded schools and the average amount of TUPE Tier 2 funding per capita.

#### Respondent Characteristics

To ascertain participants’ professional roles, the survey asked, “Which of the following BEST describes your position at the school?” The options were: teacher, paraeducator or instructional aide, counselor, social worker, school psychologist, school nurse, school administrator, campus security or campus supervisor, school support or office staff, tobacco prevention education (TUPE) advisor, coordinator, or specialist, and other. To reduce the number of categories, the analysis recategorized these as “teaching staff,” “counseling student services,” “school administration,” “TUPE staff,” and “other.” To assess length of service, the survey asked, “Approximately how long have you worked at this school?” Options were various ranges of years. To assess their involvement in prevention education, the survey asked, “Is it part of your role at the school to provide students with tobacco or other drug prevention education?” Options included “Yes,” “No, it is not part of my job, but I do it anyway,” “No, it is not part of my job,” and “I don’t know.” Those who reported providing prevention education were asked, “About how much of your time is spent on tobacco or other drug prevention education?” The options were various ranges of percentages.

#### Perceived Health Priorities

To assess what participants perceived to be the top health priorities in their schools, the survey asked, “What do you think are your school’s current TOP 3 health priorities?” The survey instructed them to select three of the following: “alcohol and other drug use”; “bullying, violence, and school safety”; “healthy eating”; “marijuana/‌cannabis use (including vaping marijuana)”; “mental health”; “obesity/‌overweight/healthy weight”; “physical activity”; “social media and screen time”; “sexual health”; and “tobacco use (including vaping nicotine or just flavoring).” To minimize order bias, the order of response options was randomized for each participant.

#### Training on Prevention

To assess staff training on substance use prevention, the survey asked, “During the PAST 3 YEARS, have YOU received training in the following areas? (e.g., attending webinars, workshops, conferences, etc.)” Options included the 10 school health priorities listed above. For this study, however, only the responses on tobacco, marijuana, and alcohol or other drugs were analyzed.

#### Tobacco Prevention Activities

To assess staff perceptions of tobacco prevention activities offered at their schools, the survey asked, “In the last 12 months, did YOUR school participate in any of the following activities to address tobacco?” with the options Red Ribbon Week, Great American Smokeout, and Kick Butts Day. The survey also asked, “In the last 12 months, did YOUR school offer any of the following activities to address tobacco?” Options included: “alternative to suspension programs (for example, YVAPE)”; “groups to help students quit smoking or vaping”; “guest presentations, e.g., from outside organization”; “health fairs”; “out of school clubs to prevent tobacco use”; “parental education on tobacco use”; “referrals to Kick It California (formerly the California Smokers’ Helpline, 1-800 No Butts),” and “student-led or initiated events targeting tobacco use.” For the study, we also calculated the proportion of respondents reporting that their school offered at least 3 activities on this list.

### Data Analysis

For the analysis of TUPE funding distribution, schools receiving Tier 2 awards in the 2020–2023 funding cycle were grouped by region, using the same definitions of regions as in the 2019–2020 CSTS, a well-established statewide school survey (Zhu et al., 2020, 2021). The highest level of geographic analysis in CSTS and its successor, the California Youth Tobacco Survey, divides California into four regions: Northern, Greater Bay, Central, and Southern (Zhu et al., 2020, 2021). A weighted average of current tobacco use prevalence for students in grades 8, 10, and 12 was computed for each of the four regions (Zhu et al., 2021). These data were then compared against the distribution of TUPE funding in the four regions. The number of students for TUPE-funded schools was first calculated using CDE’s funding information (California Department of Education, 2021). The proportion of students in TUPE-funded schools was then calculated by dividing the number attending TUPE Tier 2-funded schools in a region by the total enrollment for the region (California Department of Education, 2021, 2024). Similarly, funding per capita was calculated by dividing total annual Tier 2 funding in a region by total enrollment for the region (California Department of Education, 2021, 2024).

For the analysis of CETS data, schools were categorized in two groups: those whose districts received TUPE Tier 2 awards for 2020–2023 were considered TUPE-funded schools and all others were considered non-TUPE schools. Descriptive statistics were used to compare respondent characteristics. Results for school health priorities, substance use prevention training, and tobacco prevention activities were weighted by schools’ enrollment size (California Department of Education, 2024) and presented with 95% confidence intervals. In addition, multiple logistic regressions controlling for respondent characteristics were conducted to confirm significant results. Analyses were conducted using SAS 9.4.

## Results

Table 1 presents information about the distribution of TUPE funding. Column 1 shows the number of enrolled students in each region. Column 2 shows rates of current tobacco use in 2019–2020. At 13.8%, the Northern region had the highest prevalence rate, significantly higher than other regions. Next in order were the Greater Bay region (10.3%), Southern region (9.0%), and Central region (8.9%). Column 3 shows the proportion of students in TUPE-funded schools. The Northern and Greater Bay regions had the highest proportions of students in TUPE-funded schools, at 57.7% and 56.3%, respectively, while the Central and Southern regions had smaller proportions, at 37.0% and 36.8%, respectively. Column 4 shows funding per capita. The Northern and Greater Bay regions received $9.89 and $9.74 per capita, respectively, while the Central and Southern regions received $6.08 and $6.39, respectively.

**Table 1.** Tobacco Use Prevalence and TUPE Funding Level by Four Regions in California.

Region	Enrollment^a^	Currently use tobacco^b^ % (95% CI)	Students in TUPE schools^c^ %	Funding per capita^d^ $
Northern	307,520	13.8 (11.7–15.9)	57.7%	9.89
Greater Bay	673,249	10.3 (9.1–11.5)	56.3%	9.74
Central	388,825	8.9 (7.3–10.4)	37.0%	6.08
Southern	1,862,186	9.0 (7.7–10.2)	36.8%	6.39

a Total census day enrollment for grades 6–12 in the 2020–2021 school year. ^b^ Past 30-day tobacco use prevalence per the 2019–2020 California Student Tobacco Survey for grades 8, 10, and 12. ^c^ Percentage of students enrolled in TUPE Tier 2-funded schools in the 2020–2021 school year. ^d^ Total amount of TUPE Tier 2 funds awarded for the 2020–2021 school year divided by total enrollment.

Table 2 displays survey respondent characteristics by whether their schools were TUPE-funded. Most respondents were teachers, with a slightly higher proportion in TUPE schools than in non-TUPE schools. Respondents in non-TUPE schools were slightly more often counselors and administrators than those in TUPE-funded schools. There was no significant difference between TUPE-funded and non-TUPE schools in the other three variables examined.

**Table 2.** California Educator Tobacco Survey Respondent Characteristics.

Survey item	TUPE	Non-TUPE
	*N* = 2,482	*N* = 3,633
	% (95% CI)	% (95% CI)
Primary role in school		
Teaching staff	81.4 (79.3–83.5)	77.5 (75.3–79.8)
Counseling and student services	7.1 (5.8–8.5)	9.7 (8.0–11.3)
School administration	5.4 (4.0–6.8)	6.9 (5.5–8.3)
TUPE staff	0.4 (0.1–0.6)	0.1 (0.0–0.2)
Other	5.7 (4.3–7.0)	5.8 (4.8–6.8)
Length of service		
<1 year	9.2 (7.6–10.9)	8.0 (6.8–9.2)
1–5 years	28.5 (26.1–31.0)	29.8 (27.5–32.0)
6–10 years	22.0 (19.8–24.2)	23.3 (21.4–25.2)
>10 years	40.2 (37.5–43.0)	38.9 (36.5–41.4)
Role involves prevention education		
Yes	17.2 (15.1–19.2)	14.9 (13.2–16.5)
Not part of role, but do it anyway	28.9 (26.6–31.2)	28.4 (26.4–30.4)
No	52.1 (49.6–54.7)	54.7 (52.5–56.9)
Don’t know	1.8 (1.3–2.4)	2.0 (1.5–2.5)
Time spent on prevention education^a^		
≤10%	82.0 (79.0–85.0)	83.2 (80.6–85.7)
11%–25%	14.4 (11.7–17.0)	14.2 (11.8–16.7)
>25%	3.6 (2.0–5.3)	2.6 (1.5–3.7)

a Only those who reported providing prevention education were asked this question.

Table 3 shows respondents’ perceptions of the top health priorities in their schools. The percentages in each column add up to 300% because the survey asked respondents to select the top three priorities. Mental health was selected most often by respondents from both TUPE-funded and non-TUPE schools. In fact, the top four choices were the same for both TUPE-funded and non-TUPE schools: mental health, bullying/‌violence/‌school safety, social media and screen time, and marijuana/cannabis use. In fifth and sixth place, respondents from TUPE-funded schools chose tobacco use over alcohol and other drug use, while non-TUPE respondents chose the opposite. The switch was statistically detectable, with 28.0% of respondents from TUPE-funded schools selecting tobacco use compared with 22.6% of non-TUPE respondents (*p* < .005). In both TUPE-funded and non-TUPE schools, tobacco, alcohol, and other drug use were ranked as higher priorities than physical activity, sexual health, healthy eating, and obesity/‌overweight/healthy weight.

**Table 3.** Perceived Health Priorities in Schools by TUPE Funding Status.

Survey item	TUPE	Non-TUPE
	*N* = 2,482	*N* = 3,633
	% (95% CI)	% (95% CI)
School’s top health priorities^a^		
Mental health	83.3 (81.2–85.5)	83.0 (81.3–84.7)
Bullying, violence, school safety	59.1 (56.4–61.8)	61.5 (59.3–63.7)
Social media, screen time	42.2 (39.5–44.9)	39.9 (37.7–42.2)
Marijuana use	38.4 (35.6–41.1)	38.4 (36.1–40.8)
Tobacco use	28.0 (25.4–30.6)	22.6 (20.5–24.8)
Alcohol, other drug use	22.0 (19.6–24.4)	24.7 (22.7–26.7)
Physical activity	10.2 (8.3–12.1)	10.6 (9.2–11.9)
Sexual health	7.2 (5.8–8.6)	7.6 (6.4–8.8)
Healthy eating	5.8 (4.6–6.9)	7.4 (6.3–8.5)
Obesity, overweight	3.8 (2.9–4.6)	4.2 (3.4–5.1)

a 3 selections were required, so the percentages in each column add up to approximately 300%. Due to independent rounding, they may not add up to exactly 300%.

Table 4 shows the proportion of respondents who received training on tobacco, marijuana, or alcohol and other drug use in the past 3 years. Those from TUPE-funded schools were more likely to receive tobacco training (29.0% vs. 24.6%; *p* < .01). A multiple regression model controlling for the respondent characteristics in Table 1 found an odds ratio (OR) of 1.4 (95% confidence interval [CI] = [1.1, 1.8]), confirming that the difference was statistically significant. In contrast, there was no significant difference regarding training on alcohol and other drug use (24.6% vs. 23.7%; *p* = .56) or on marijuana use (27.2% vs. 25.3%; *p* = .25).

**Table 4.** Received Training for Substance Use Prevention by TUPE Funding Status.

Survey item	TUPE	Non-TUPE
	*N* = 2,482	*N* = 3,633
	% (95% CI)	% (95% CI)
Training received in the past 3 years		
Tobacco use	29.0 (26.4–31.7)	24.6 (22.5–26.7)
Marijuana use	27.2 (24.8–29.7)	25.3 (23.1–27.5)
Alcohol, other drug use	24.6 (22.2–27.1)	23.7 (21.6–25.8)

Table 5 shows which events respondents recalled their schools participating in over the past 12 months. They first were asked if their schools participated in Red Ribbon Week, the Great American Smokeout, and Kick Butts Day. Red Ribbon Week is a general health activity which may or may not include tobacco prevention, whereas the Great American Smokeout and Kick Butts Day both focus on tobacco prevention. High percentages of both TUPE-funded and non-TUPE schools conducted Red Ribbon Week, with no significant difference between them (40.1% vs. 37.7%; *p* = .32). In contrast, TUPE-funded schools were more likely to participate in the two tobacco-specific events, the Great American Smokeout (9.5% vs. 5.8%; *p* < .005) and Kick Butts Day (5.0% vs. 1.3%; *p* < .001).

**Table 5.** Tobacco Prevention Activities at School by TUPE Funding Status.

Survey item	TUPE	Non-TUPE
	*N* = 2,482	*N* = 3,633
	% (95% CI)	% (95% CI)
School participation in events		
Red Ribbon Week	40.1 (36.4–43.8)	37.7 (34.6–40.7)
Great American Smokeout	9.5 (7.5–11.4)	5.8 (4.7–6.9)
Kick Butts Day	5.0 (3.7–6.3)	1.3 (0.8–1.8)
Tobacco prevention activities offered		
Student-led or -initiated events	20.9 (18.4–23.5)	9.4 (8.0–10.8)
Alternative to suspension programs	19.6 (17.3–22.0)	15.9 (14.1–17.8)
Parental education on tobacco use	17.6 (15.4–19.9)	16.9 (15.0–18.9)
Guest presentations	16.3 (14.3–18.4)	14.9 (13.2–16.6)
Groups to help students quit	16.1 (13.8–18.3)	12.4 (10.7–14.0)
Health fairs	13.9 (11.8–15.9)	13.6 (11.8–15.3)
Out of school clubs	13.7 (11.5–15.8)	6.4 (5.4–7.5)
Referrals to state quitline	6.0 (4.5–7.4)	2.8 (2.1–3.4)
Offered at least three of the listed activities^a^	19.3 (17.0–21.6)	12.7 (11.0–14.4)

a Combination of the eight items in the second question (i.e., excluding Red Ribbon Week, Great American Smokeout, and Kick Butts Day).

Table 5 also shows what other activities respondents recalled their schools offering to address tobacco. There was no significant difference between TUPE-funded and non-TUPE schools in offering parental education (17.6% vs 16.9%; *p* = .65), guest presentations (16.3% vs. 14.9%; *p* = .28), or health fairs (13.9% vs. 13.6%; *p* = .83). In contrast, TUPE-funded schools were significantly more likely to offer student-led or -initiated events (20.9% vs. 9.4%; *p* < .001), alternative to suspension programs (19.6% vs. 15.9%; *p* < .05), groups to help students quit (16.1% vs. 12.4%; *p* < .05), out of school clubs (13.7% vs. 6.4%; *p* < .001), and referrals to a statewide cessation resource (6.0% vs. 2.8%; *p* < .001). When responses on all eight options in this list were combined, TUPE-funded schools were significantly more likely than non-TUPE schools to offer at least three types of activities (19.3% vs. 12.7%; *p* < .001). A multiple regression model controlling for the respondent characteristics in Table 1 found that TUPE-funded schools were twice as likely to reach this threshold (OR = 2.1; 95% CI = [1.6, 2.8]).

## Discussion

This study found that regions of California with higher rates of student tobacco use received more funding for tobacco use prevention education than regions with lower student use rates. Among the four regions investigated, funding per capita followed the same rank order as that of youth tobacco use prevalence rates, an encouraging sign for equity considerations. Notably, the mostly rural Northern region of California, where more than one in eight students used tobacco, also had the greatest proportion of students in TUPE-funded schools and secured the most TUPE funding per capita.

Most respondents to the California Educator Tobacco Survey did not perceive tobacco use as a top health priority in their schools. They consistently ranked four other issues higher than tobacco, including marijuana use (Table 2). This was not entirely surprising, as marijuana use has become more prevalent than tobacco use among California students (Zhu et al., 2020). Nevertheless, it is telling that even staff of TUPE-funded schools perceived marijuana as a higher priority than tobacco. This suggests that motivating additional school districts to establish tobacco prevention programs may be challenging due to competing priorities that can make youth tobacco use seem like a less urgent problem.

TUPE funding appeared to increase tobacco prevention activity, as shown in two key measures. First, TUPE-funded school staff were statistically more likely than non-TUPE school staff to report that they received training on tobacco prevention, whereas no such difference was detected for training on marijuana, alcohol, and other drug prevention. It could be expected that most schools would address the substances together when training staff, given the close association in students’ use of various substances, so it is notable that such a differential result emerged. Second, TUPE-funded schools did not have a higher level of prevention activity than non-TUPE schools across the board. For example, Red Ribbon Week, a popular school activity that aims to prevent the use of a range of substances, including tobacco, alcohol, and other drugs, was no more likely to occur in TUPE-funded schools, whereas tobacco-specific activities such as the Great American Smokeout and Kick Butts Day *were* more likely to occur in TUPE-funded schools.

Moreover, while TUPE-funded schools were no more likely to adopt prevention practices such as inviting guest speakers and conducting health fairs, differences emerged regarding certain other activities, such as student-led or -‍initiated events, alternative to suspension programs, out of school clubs, and groups to help students quit tobacco. These generally resource-intensive activities focus on engaging youth on tobacco and align well with the TUPE program’s emphasis on providing a full suite of prevention, intervention, cessation, and youth development services (California Department of Education, Tobacco-Use Prevention Education Office, 2020). It is in these relatively resource-intensive and tobacco-specific activities that TUPE-funded schools outperformed their non-TUPE counterparts. Taken together, these results suggest that TUPE funding was at least partly responsible for the observed increase in tobacco training activity in TUPE-funded schools, and that TUPE funding facilitated the implementation of tobacco-focused activities and services, which was limited in non-TUPE schools.

In the context of previous evaluations of California’s TUPE program, this study highlights a tobacco prevention program that appears to have evolved and matured over time. An early study in the first decade of the program found little evidence that tobacco prevention curricula were widely implemented or influential with students; it also found that TUPE funding per capita had already been decreasing for several years running (Distefan et al., 2000). A decade later, a study using a nested school-longitudinal design found that TUPE did appear to reduce student tobacco use over time, although other contextual factors such as the socioeconomic characteristics of schools were moderating influences (Park et al., 2010). A more recent study found that TUPE-funded schools were more likely to prioritize tobacco use prevention, train staff to address tobacco use among students, and provide tobacco-specific prevention programs than non-TUPE schools (McMenamin et al., 2018). The current study confirms these more recent findings, and as the first evaluation of TUPE since the infusion of new funding from Proposition 56, documents that the distribution of funding was aligned with regional differences in youth tobacco use.

### Limitations and Strengths

This study had limitations. First, despite incentives for participation, the individual-level survey response rate was only 6.3%. This reflects in part the challenge of assembling an accurate contact list for staff of numerous schools across a large state, and the fact that some schools filtered out emails sent to their staff. These challenges notwithstanding, the school-level response rate was much higher, 79.3%. Second, it is not possible to determine to what extent the observed differences between TUPE-funded and non-TUPE schools were due to funding, although, as discussed above, the results exhibited a pattern suggesting that the differences can be attributed at least partly to funding, and multiple logistical regressions confirmed that the differences in tobacco prevention activities were robust. Finally, the analyses did not account for clustering, mainly because the low number of respondents per school (*M* = 4.5) precluded a reliable estimation of intraclass correlation coefficients.

The main strength of the study was the large number of respondents (*n* = 6,115) and the large number of schools (*n* = 1,349) across California that participated in the survey, leading to a consistent pattern observed between TUPE-funded and non-TUPE schools.

### Implications for Policy and Practice

California’s long-standing TUPE Program has a dedicated funding stream from tobacco tax revenues (California Department of Education, 2023), but the available funding is insufficient to cover all public schools in the state. It is encouraging, therefore, that the allocation of funding observed in this study was greatest in regions with the highest youth tobacco use rates. This is likely due in part to outreach conducted by the California Department of Education to encourage applications from rural, high-prevalence areas of the state. This may be an important consideration for jurisdictions seeking to reduce geographic disparities in youth tobacco use rates.

TUPE funding apparently helped raise the profile of tobacco in school districts that received it, but the overall low prioritization of tobacco use among school health issues remains a challenge. Future tobacco prevention efforts may aim for closer integration with other student health programs, such as those for mental health and marijuana use, which rank higher as school health priorities. Integration of programs may help pool resources, create synergy, and reach more students.

## Conclusion

This study shows that funding from the California Department of Education Tobacco-Use Prevention Education Program in the 2020–2023 grant cycle was associated with increased tobacco training and prevention activity in funded schools compared with non-funded schools. The study contributes to efforts to evaluate school-based tobacco prevention programs by demonstrating the value of school staff surveys in assessing the implementation of tobacco prevention programming.
